# P-565. A Prospective and Retrospective Observational Study of Multidrug-Resistant Patient Outcomes with and without Ibalizumab in a Real-World Setting: United States (PROMISE-US) Study Design and Baseline Characteristics

**DOI:** 10.1093/ofid/ofae631.763

**Published:** 2025-01-29

**Authors:** Princy N Kumar, Charlotte-Paige M Rolle, Brinda Emu, Zachary Henry, Claudia Martorell, John E McKinnon, Jihad Slim, R Brandon Cash, Kaitlin Anstett

**Affiliations:** Georgetown University Medical Center, Washington, District of Columbia; Orlando Immunology Center; Emory Rollins School of Public Health, Orlando, Florida; Yale University, New Haven, CT; AHF Northpoint Healthcare Center, Fort Lauderdale, Florida; The Research Institute, springfield, Massachusetts; Division of Infectious Diseases, Department of Medicine, Medical University of South Carolina, Charleston, South Carolina; Saint Michael’s Medical Center, Newark, NJ, USA, Newark, New Jersey; Theratechnologies, Inc, Montreal, Quebec, Canada; Theratechnologies Inc., Canada

## Abstract

**Background:**

Multidrug resistance arises usually in people with HIV (PWH) with extensive prior exposure to antiretrovirals (ARVs). There is a need for further understanding of factors that impact the maintenance of virologic suppression in heavily treatment experienced (HTE) PWH in a real-world setting.

**Table 1:**
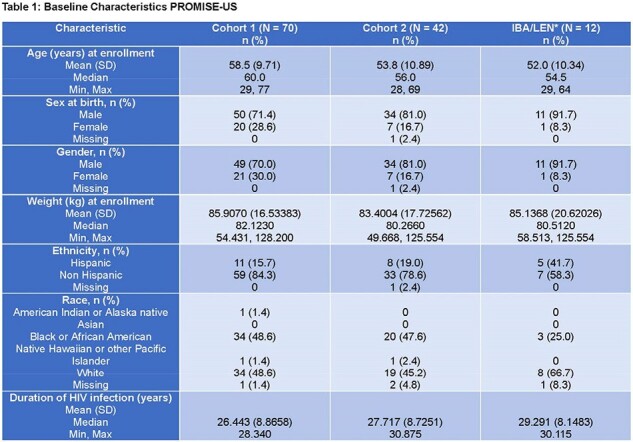
Baseline Characteristics PROMISE-US 1/2 * Any regimen including the two drugs and may have additional ARVs in the OBR ** cGSS was calculated as per Gonzalez-Serna et al. Journal of Antimicrobial Chemotherapy, 2017, P. 496 –503, https://doi.org/10.1093/jac/dkw455. Because commercial resistance testing is not available for ibalizumab, fostemsavir, or lenacapavir, all 3 agents are always considered fully active. *** Adverse events were only collected for subjects in Cohort 2. ARV, antiretroviral; cGSS, combined genotypic sensitivity score; FTR, fostemsavir; IBA, ibalizumab; INSTI, integrase strand transfer inhibitor; LEN, lenacapavir; MVC, maraviroc; NNRTI, non-nucleotide reverse transcriptase inhibitor; NRTI, nucleotide reverse transcriptase inhibitor; OBR, optimized background regimen; PI, protease inhibitor; RNA, ribonucleic acid.

**Methods:**

PROMISE-US (CT.gov Identifier: NCT05388474) is a phase 4 multicenter, retrospective and prospective, observational, non-interventional registry study. The primary objective is to evaluate the long-term efficacy and durability of ibalizumab in combination with other ARVs by comparing the clinical outcomes of patients receiving ibalizumab treatment (Cohort 2; C2) vs. matched patients not receiving ibalizumab (Cohort 1; C1). This abstract describes the study design and baseline characteristics of the first 100 patients enrolled. Baseline is defined as the date of the latest treatment change event (the start date of ibalizumab in C2; the start date of standard of care in C1).

Table 1: Baseline Characteristics PROMISE-US 2/2

**Figure:**
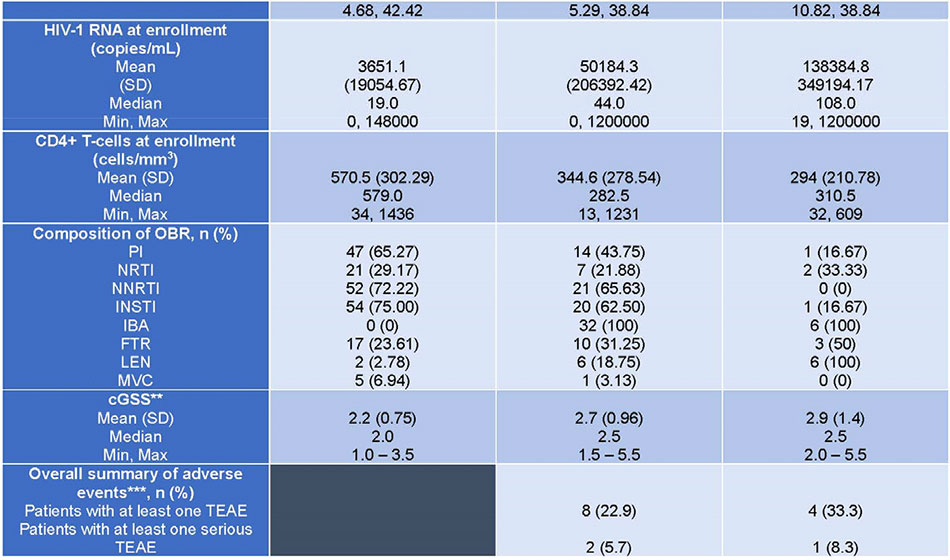

*Any regimen including the two drugs and may have additional ARVs in the OBR

**cGSS was calculated as per Gonzalez-Serna et al. Journal of Antimicrobial Chemotherapy, 2017, P. 496 –503, https://doi.org/10.1093/jac/dkw455. Because commercial resistance testing is not available for ibalizumab, fostemsavir, or lenacapavir, all 3 agents are always considered fully active.

***Adverse events were only collected for subjects in Cohort 2.

ARV, antiretroviral; cGSS, combined genotypic sensitivity score; FTR, fostemsavir; IBA, ibalizumab; INSTI, integrase strand transfer inhibitor; LEN, lenacapavir; MVC, maraviroc; NNRTI, non-nucleotide reverse transcriptase inhibitor; NRTI, nucleotide reverse transcriptase inhibitor; OBR, optimized background regimen; PI, protease inhibitor; RNA, ribonucleic acid.

**Results:**

By 8-Nov-2023, a total of 114 subjects were enrolled; 70 in 1, 44 in C2. Baseline characteristics, including race, ethnicity, sex, gender, and time since diagnosis, were well matched between both cohorts. The use of ibalizumab and the combination of ibalizumab plus lenacapavir was associated with HTE patients with higher HIV viral loads and declining CD4 T cells (p-values of 0.0629 and 0.0001, respectively, for C2 vs. C1). Combined genotypic sensitivity scores (cGSSs) were comparable between the two cohorts (Table 1). However, since commercial resistance testing is not available for all newer agents, which are considered fully active, this measure may overestimate regimen sensitivity in C2 subjects who were more likely to have these in their regimens. 80% of C2 subjects had been on ibalizumab for greater than 12 months. Ibalizumab was well-tolerated with no infusion reactions reported.

**Conclusion:**

Ibalizumab was more frequently selected for use in advanced HTE patients with lower CD4 cell counts and higher viral loads than other regimens. Through better understanding of HTE patients’ treatments and long-term use of ibalizumab, this study will help further characterize the efficacy and safety profile of the agents used in combination for this population with high unmet need to help guide treatment selection.

**Disclosures:**

**Princy N. Kumar, MD**, Gilead: Advisor/Consultant|Gilead: Grant/Research Support|Gilead: Stocks/Bonds (Public Company)|Johnson & Johnson: Stocks/Bonds (Public Company)|Merck: Advisor/Consultant|Merck: Grant/Research Support|Merck: Stocks/Bonds (Public Company)|Moderna: Stocks/Bonds (Public Company)|Pfizer: Stocks/Bonds (Public Company)|Theratechnologies: Grant/Research Support|ViiV/GSK: Advisor/Consultant|ViiV/GSK: Grant/Research Support|ViiV/GSK: Stocks/Bonds (Public Company) **Charlotte-Paige M. Rolle, MD, MPH**, Gilead Sciences: Advisor/Consultant|Gilead Sciences: Grant/Research Support|Gilead Sciences: Honoraria|MSD: Grant/Research Support|ViiV Healthcare: Advisor/Consultant|ViiV Healthcare: Grant/Research Support|ViiV Healthcare: Honoraria **Brinda Emu, MD**, Genentech/Roche: Advisor/Consultant|Theratechologies: Advisor/Consultant **Zachary Henry, MD**, gilead: Advisor/Consultant|theratechnology: Advisor/Consultant **Claudia Martorell, MD**, Gilead Sciences: Grant/Research Support|Theratechnologies: Grant/Research Support|ViiV: Grant/Research Support **Jihad Slim, MD, FACP**, AbbVie: Grant/Research Support|AbbVie: Honoraria|AbbVie: Speaker Bureau|Gilead Sciences, Inc.: Grant/Research Support|Gilead Sciences, Inc.: Honoraria|Gilead Sciences, Inc.: Speaker Bureau|Merck: Grant/Research Support|Merck: Honoraria|Merck: Speaker Bureau|Theratechnologies: Advisor/Consultant|Theratechnologies: Honoraria|ViiV Healthcare: Advisor/Consultant|ViiV Healthcare: Grant/Research Support|ViiV Healthcare: Speaker Bureau **R Brandon Cash, PharmD**, Theratechnologies: Employee **Kaitlin Anstett, PhD**, Theratechnologies Inc.: Employee

